# hnRNPU in Sertoli cells cooperates with WT1 and is essential for testicular development by modulating transcriptional factors *Sox8/9*

**DOI:** 10.7150/thno.66819

**Published:** 2021-10-25

**Authors:** Yujiao Wen, Xixiang Ma, Xiaoli Wang, Fengli Wang, Juan Dong, Yanqing Wu, Chunyu Lv, Kuan Liu, Yan Zhang, Zhibing Zhang, Shuiqiao Yuan

**Affiliations:** 1 Institute of Reproductive Health, Tongji Medical College, Huazhong University of Science and Technology, Wuhan, Hubei 430030, China.; 2 Department of Obstetrics & Gynecology, Wayne State University, Detroit, MI 48201, USA.; 3 Department of Physiology, Wayne State University, Detroit, MI 48201, USA.; 4 Shenzhen Huazhong University of Science and Technology Research Institute, Shenzhen, Guangdong 518057, China.

**Keywords:** hnRNPU, Sertoli cells, Testicular development, Transcription factor, Mice

## Abstract

**Background:** Sertoli cells are essential regulators of testicular fate in the differentiating gonad; however, its role and underlying molecular mechanism of regulating testicular development in prepubertal testes are poorly understood. Although several critical regulatory factors of Sertoli cell development and function have been identified, identifying extrinsic factors that regulate gonocyte proliferation and migration processes during neonatal testis development remains largely unknown.

**Methods:** We used the Sertoli cell-specific conditional knockout strategy (Cre/Loxp) in mice and molecular biological analyses (Luciferase assay, ChIP-qPCR, RNA-Seq, etc.) in vitro and in vivo to study the physiological roles of hnRNPU in Sertoli cells on regulating testicular development in prepubertal testes.

**Results:** We identified a co-transcription factor, hnRNPU, which is highly expressed in mouse and human Sertoli cells and required for neonatal Sertoli cell and pre-pubertal testicular development. Conditional knockout of hnRNPU in murine Sertoli cells leads to severe testicular atrophy and male sterility, characterized by rapid depletion of both Sertoli cells and germ cells and failure of spermatogonia proliferation and migration during pre-pubertal testicular development. At molecular levels, we found that hnRNPU interacts with two Sertoli cell markers WT1 and SOX9, and enhances the expression of two transcriptional factors, *Sox8* and *Sox9,* in Sertoli cells by directly binding to their promoter regions. Further RNA-Seq and bioinformatics analyses revealed the transcriptome-wide of key genes essential for Sertoli cell and germ cell fate control, such as biological adhesion, proliferation and migration, were deregulated in Sertoli cell-specific hnRNPU mutant testes.

**Conclusion:** Our findings demonstrate an essential role of hnRNPU in Sertoli cells for prepubertal testicular development and testis microenvironment maintenance and define a new insight for our understanding of male infertility therapy.

## Introduction

In sexual reproduction, paternal spermatozoa are one bridge of genetic and epigenetic information transmission to the next generation. These specialized cells are formed by spermatogonial stem cells (SSCs) through mitosis, meiosis and spermiogenesis, collectively called spermatogenesis. The foundational SSC pool is the basis of sustained spermatogenesis 1, 2. The niche microenvironment that supports gonocyte development and establishment of the foundational SSCs pool is mainly contributed by Sertoli cells, a specialized epithelial cell that surrounds the male germline 3. Sertoli cells are the only somatic cell within the seminiferous tubules (or testis cords). Through support, nutrition, and secretion, Sertoli cells not only take part in regulating male sex differentiation in mammalian embryonic development but are also necessary for testicular development and spermatogenesis 4, 5. During the early stage of mouse embryonic development, the origin of pre-Sertoli precursor cells in the gonadal primordium is dependent on the activation of the Y-linked sex-determining gene *Sry* around embryonic day 10.5 (E10.5). Subsequently, upregulated sex-determining genes promote the bipotential gonad differentiation into XY gonad, then the differentiated pre-Sertoli cells in the XY gonad orchestrate the organization of the testis cords with prospermatogonia 6-9. Subsequently, male germline establishment requires prospermatogonia (Prospg) to migrate from the middle of the testis cord to the basement membrane and resume the mitotic cell cycle during neonatal development of the testis (postnatal day 0 to 3) 10. These events are regulated by the testicular microenvironment outside the Prospg, and Sertoli cells are considered to play an important role in the maintenance of testicular microenvironment 11.

Several important regulatory factors of Sertoli cell development and function have been identified. Nuclear transcription factor Wilms' tumor 1 (WT1), which is encoded by the Wilms' tumor suppressor gene *Wt1*, is essential for Sertoli cell differentiation, testis development, and normal Sertoli cell function in adult testes 12, 13. Many critical genes are likely activated by *Wt1* in the gonad, such as NR5A1 and CITED2 14, 15. Conditional knockout of *Wt1* in the Sertoli cell leads to aberrant development of the testis cord 16. The SOX E transcription factors *Sox8/9* are two important regulatory factors that concerted and redundantly regulate testis cord formation and the development of the seminiferous tubules. *Sox8/9* double mutants exhibited a severe testicular development cease and abnormal Sertoli cell development phenotypes for downregulation of some cell adhesion molecules and structural components of the basal lamina 17, 18. However, identifying extrinsic factors that induce germ cell migration and cell cycle recovery during neonatal testis development is challenging because they coincide. Previous studies showed that transforming growth factor-beta family proteins (TGFβ1, TGFβ2 and Activin A) secreted by Sertoli cells participate in inducing and maintaining the cell cycle arrest of prospermatogonia during embryonic and early neonatal period 19, 20. After birth, the decreased secretion of these factors and the increase in mitogen (including FGF2 and GDNF) synergistically promote the proliferation of prospermatogonia 21. Increasing evidence showed that some Sertoli cell-derived regulatory factors, such as TGFβ family 19, FGF2 21, KITL 22, and PDGF family 23 proteins, are required for spermatogonia development and migration. However, the molecular mechanism, especially the key factors, regulating gonocyte proliferation and migration processes during neonatal testis development remains largely unknown.

Heterogeneous nuclear ribonucleoproteins (hnRNPs) are a large family of RNA binding proteins that regulate multiple aspects of nucleic acid metabolism, including mRNA transcription, alternative splicing, stability and translational regulation 24. The largest hnRNP family member, hnRNPU, can bind to RNA through the RGG box in the glycine-rich C terminus 24, 25 and interact with transcriptional and splicing factors to regulate the processing of transcription and alternative splicing 26, 27. As an essential transcriptional regulator, hnRNPU has been demonstrated to modulate many vital biological processes. For example, loss of function variants of hnRNPU causes early-onset epilepsy and severe intellectual disability 28, 29. hnRNPU also regulates T-cell activation and postnatal heart development through pre-mRNA splicing 30, 31. However, due to the early lethality of hnRNPU knockout mice 32, the *in vivo* biological functions of hnRNPU in mammalian tissue development remain to be expounded.

In this report, we show that the hnRNPU protein is highly expressed in both mouse and human Sertoli cells and is essential for Sertoli cell development and male fertility. The loss of function of hnRNPU in mouse Sertoli cells causes complete male infertility, characterized by severe testicular atrophy, rapid depletion of both Sertoli cells and germ cells, aberrant spermatogonia development and migration during pre-pubertal testicular development. We also discovered that hnRNPU could interact with WT1 and SOX9, two Sertoli cell markers, and directly bind to the promoter regions of *Sox8* and *Sox9* to regulate Sertoli cell and testicular development. Our data, for the first time, identified a critical role of hnRNPU in Sertoli cells for maintaining the testicular microenvironment and development in pre-pubertal testes.

## Results

### Endogenous hnRNPU and WT1 interact in mouse testis

**Figure 1:**
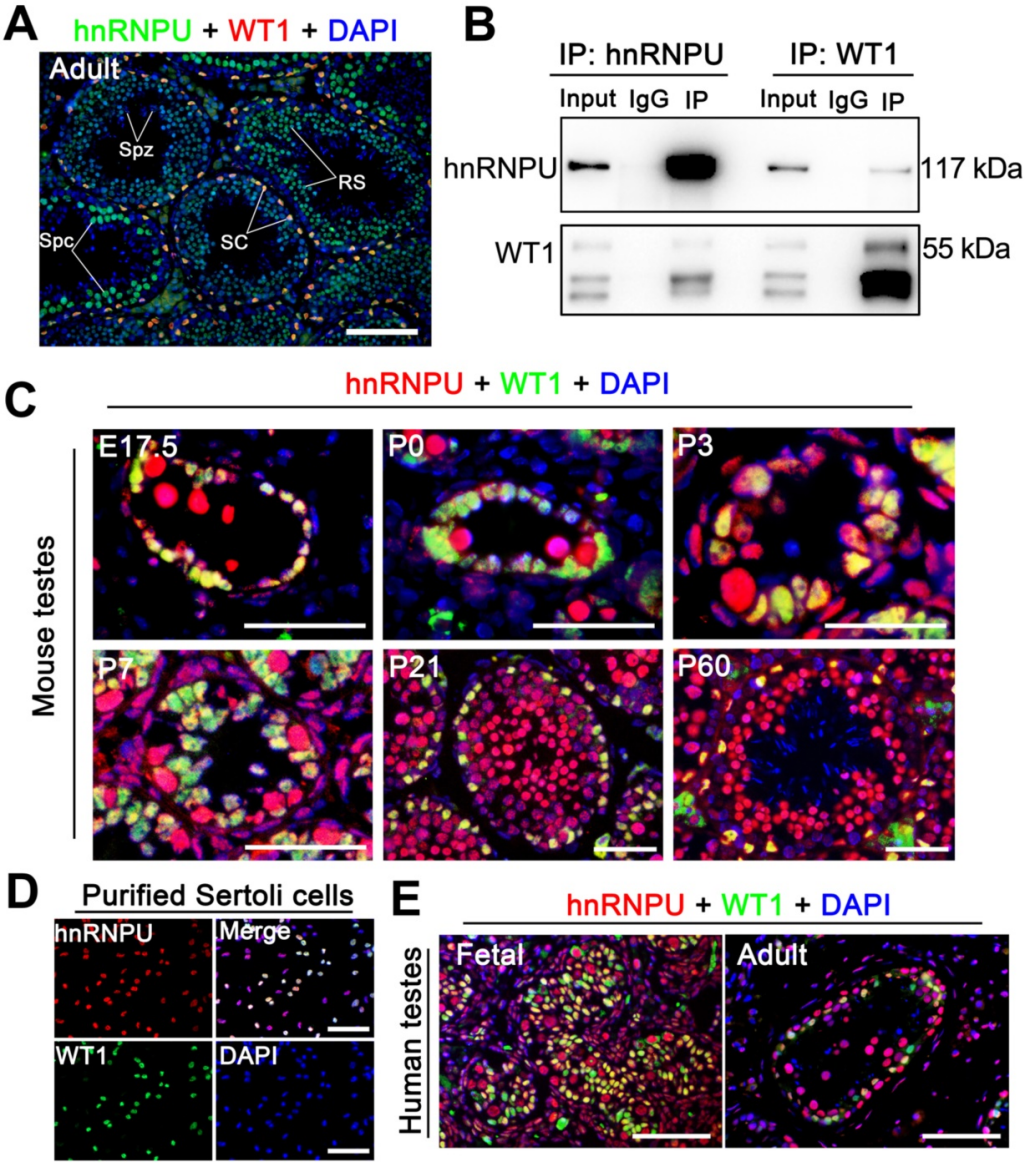
** hnRNPU localizes in mouse and human Sertoli cells and interacts with WT1 in mouse testes.** (**A**) Double immunofluorescent staining with hnRNPU (green) and WT1 (red) on adult wild-type (WT) mouse testis sections are shown. The nuclei were stained with DAPI (blue). Spz, spermatozoa; Spc, spermatocyte; SC, Sertoli cell; RS, round spermatid. Scale bars = 100 µm. (**B**) Co-immunoprecipitation assays analyze the interaction between hnRNPU and WT1 in mouse testes of postnatal day 3 (P3). Lysates of testes were immunoprecipitated with anti-hnRNPU and anti-WT1 antibodies, respectively. (**C**) Co-immunostainings of hnRNPU (red) with WT1 (green) was performed on various ages of WT mouse testis sections at embryonic day 17.5 (E17.5), P0, P3, P7, P21, and P60 are shown. The nuclei were stained with DAPI (blue). Scale bars = 50 µm. (**D**) Double immunofluorescent staining of hnRNPU (red) with WT1 (green) on isolated WT mouse Sertoli cells are shown. The nuclei were stained with DAPI (blue). Scale bars = 50 µm. (**E**) Double immunofluorescent staining with hnRNPU (red) and WT1 (green) on fetal (left) and adult (right) human testis sections are shown. The nuclei were stained with DAPI (blue). Scale bars = 50 µm.

Since hnRNPU could interact with WT1 in embryonic stem (ES) cells 33 and WT1 is specifically expressed in the Sertoli cells of the mouse testes 34, 35, we speculated that hnRNPU could interact with WT1 in mouse Sertoli cells. To test this hypothesis, we first examined the localization of hnRNPU and WT1 in adult mouse testis sections by immunofluorescence staining. The results showed that hnRNPU is expressed in both Sertoli cell and germ cell nuclei and co-localized with WT1 in Sertoli cells (**Figure 1A**), suggesting hnRNPU may interact with WT1 in Sertoli cells. To explore the possibility of the interaction between hnRNPU and WT1 in testis, we then performed immunoprecipitation (IP) using an anti-hnRNPU antibody followed by an unbiased liquid chromatography-mass spectrometry (LC/MS) analysis. The IP-LC/MS analyses showed that WT1 could be detected in hnRNPU immunoprecipitate in mouse testes (**Table S1**), indicating WT1 binds with hnRNPU in mouse testes. Reciprocal immunoprecipitations (IPs) from mouse testicular lysate by immunoblotting with the reciprocal antibody have further confirmed the interaction between hnRNPU and WT1 in mouse testes (**Figure 1B**). Together, these results suggest that hnRNPU is expressed in Sertoli cells and interacts with WT1 in mouse testis.

### hnRNPU is expressed in Sertoli cells through pre-pubertal to adult testis in both humans and mice

To precisely determine the expression profile of hnRNPU in mammals, we first examined the mRNA and protein levels in multiple organs and developmental testes of mice. The results showed that both mRNA and protein of hnRNPU were highly expressed in mouse reproductive organs, and hnRNPU was continually expressed in postnatal day 0 (P0) testes to adult testes (**Figure S1A-D**). We then analyzed the expression of hnRNPU in mouse testes at various ages by immunofluorescence staining. The results showed a high level of hnRNPU in Sertoli cells of testes from fetus (E17.5) to adulthood (**Figure 1C**). To further confirm the localization of hnRNPU in Sertoli cells, we isolated mouse Sertoli cells to stain with hnRNPU and found that hnRNPU was indeed expressed in isolated Sertoli cells (**Figure 1D**). Since multi-alignment and phylogenetic analyses of hnRNPU revealed *Hnrnpu* can encode a highly conserved protein expressed in multiple vertebrate species, including mouse, human, rat, bovine and zebrafish, etc. (**Figure S1E**), we examined the expression of hnRNPU in human Sertoli cells from fetal and adult testes by immunofluorescence staining as well. As expected, hnRNPU was highly expressed in human Sertoli cells in both fetal and adult testes (**Figure 1E**). Together, these data indicate that hnRNPU is expressed in both mouse and human Sertoli cells from fetus to adulthood and may play a role in Sertoli cell development and male fertility.

### Ablation of hnRNPU in Sertoli cells leads to severe testicular atrophy and male infertility

**Figure 2:**
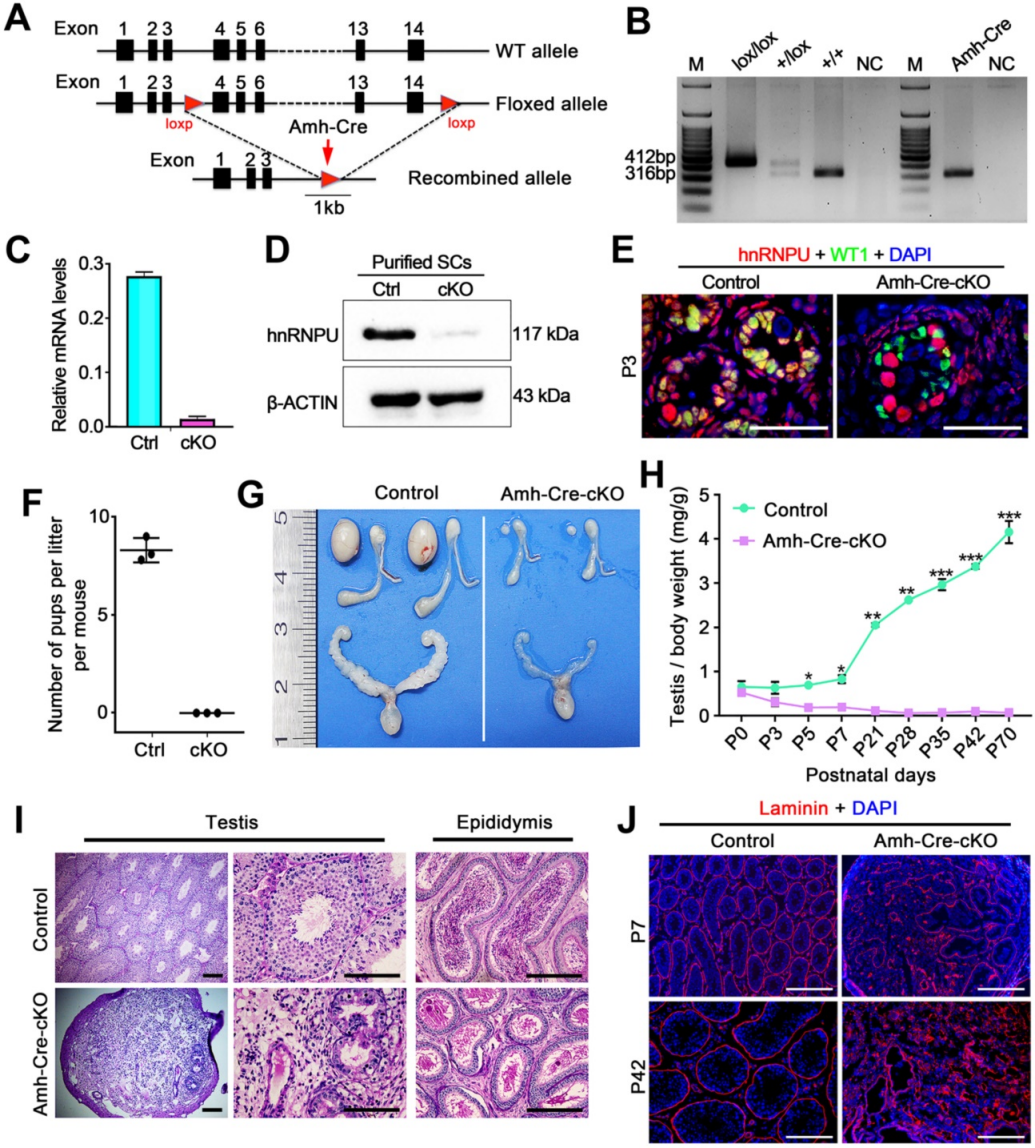
** Conditional inactivation of hnRNPU in Sertoli cells results in severe obstruction of testicular development and male infertility in mice.** (**A**) Schematic diagram showing the targeting strategy of generating a floxed *Hnrnpu* allele through homologous recombination in the murine embryonic stem cells. Red triangles represent loxP cassettes. Exons 4 to 14 will be deleted after *Amh-Cre* mediated recombination. (**B**) Representative PCR genotyping results showing the floxed (lox) and the WT (+) alleles can be detected at 412 bp and 316 bp bands, respectively. NC, non-template control. (**C**) RT-qPCR analyses showing *Hnrnpu* mRNA level was nearly undetectable in isolated Amh-Cre-cKO Sertoli cells. Data are presented as mean ± SEM, n = 3. (**D**) Western blot analyses of hnRNPU protein level in Sertoli cells (SCs) isolated from control and Amh-Cre-cKO testes. β-ACTIN served as a loading control. (**E**) Representative co-immunofluorescent images of hnRNPU (red) and WT1 (green) in control and Amh-Cre-cKO testis sections at P3 are shown. Scale bars = 50 µm. (**F**) The histogram shows the average number of pups per litter produced from control and Amh-Cre-cKO male mice. Data are presented as mean ± SEM, n = 5. (**G**) Gross morphology of the testis, the epididymis, and the seminal vesicle from adult control and Amh-Cre-cKO male mice. (**H**) Testis growth curve shows the Amh-Cre-cKO mouse testes were significantly decreased from P5. Data are presented as mean ± SEM, n = 3. *P < 0.05, **P < 0.01, and ***P < 0.001 by Student's* t-*test. (**I**) Periodic Acid-Schiff (PAS) staining shows the histology of testis and epididymis sections from adult control and Amh-Cre-cKO male mice. Left panels indicate testis histology of control and Amh-Cre-cKO mice. Many degenerated tubules containing aberrant Sertoli cell-like or germ cell-like cells were seen in the seminiferous tubules from the Amh-Cre-cKO mice. Right panels show cauda epididymis was completely lacking spermatozoa in Amh-Cre-cKO mice. Scale bars = 100 µm. (**J**) Immunofluorescence staining with Laminin (Red) in control and Amh-Cre-cKO testes at P7 and P42 are shown, respectively. The nuclei were stained with DAPI (blue). Scale bars = 100 µm.

Since hnRNPU was found to be highly expressed in Sertoli cells (SCs), we next generated SC-specific conditional knockout (cKO) mice by crossing *Hnrnpu^flox/flox^* mice 31 with the *Amh-Cre* mouse line 36 to dissect the function of hnRNPU in Sertoli cell development and male fertility. The floxed *Hnrnpu* allele contains loxP sites flanking exons 4-14, and *Amh-Cre* mediated recombination leads to an *Hnrnpu* null allele in Sertoli cells (**Figure 2A**). Mice of genotype *Amh-Cre; Hnrnpu^flox/flox^* are herein designated Amh-Cre-cKO, and controls refer to age-matched *Hnrnpu^flox/flox^* or wild-type (WT) mice unless otherwise stated (**Figure 2B**). Both mRNA and protein levels of hnRNPU were almost undetectable in Amh-Cre-cKO purified SCs, whereas controls showed high levels (**Figure 2C-D**), indicating that hnRNPU was inactivated specifically in mouse Sertoli cells. Further co-staining of hnRNPU with WT1 (a Sertoli cell marker) in control and Amh-Cre-cKO testis sections at postnatal day 3 (P3) confirmed a high Cre recombination efficiency (~100%) (**Figure 2E**). These data suggest that, in Amh-Cre-cKO male mice, the *Hnrnpu* gene was successfully ablated in Sertoli cells.

To investigate the fertility of Amh-Cre-cKO mice, we bred the Amh-Cre-cKO males with fertility-proven WT females for 6 months. Amh-Cre-cKO males never produced any pups during the fecundity test, suggesting that Amh-Cre-cKO males are sterile (**Figure 2F**). Consistent with this infertile phenotype, testis size from Amh-Cre-cKO mice was significantly smaller than their control littermates, ~5% of controls (**Figure 2G**). The ratio of testis weight/body weight of Amh-Cre-cKO mice was obviously decreased at various ages starting from P5 to P70 compared with controls (**Figure 2H**). Histological analyses showed that adult Amh-Cre-cKO testes had many degenerated tubules containing aberrant Sertoli cell-like or germ cell-like cells, whereas robust spermatogenesis was observed in control testes (**Figure 2I**). In control mice, mature spermatozoa were observed in the cauda of the epididymis but not in Amh-Cre-cKO mice (**Figure 2I**). Consistent with this histological morphology, immunofluorescence staining of Laminin (a basal membrane marker) showed a disrupted and diffuse seminiferous tubular basement membrane in Amh-Cre-cKO testes at P7 and P42 (**Figure 2J**). TUNEL assay revealed that the number of apoptotic cells in Amh-Cre-cKO testes at P3 to P7 was increased significantly compared to that of control testes (**Figure S2A-B**). Together, these data indicate that conditional ablation of *Hnrnpu* in mouse Sertoli cells causes severe testicular atrophy and disrupted spermatogenesis, thereby inducing male sterility in adulthood.

### hnRNPU in Sertoli cells is required for testicular development and pre-pubertal Sertoli cell development

**Figure 3:**
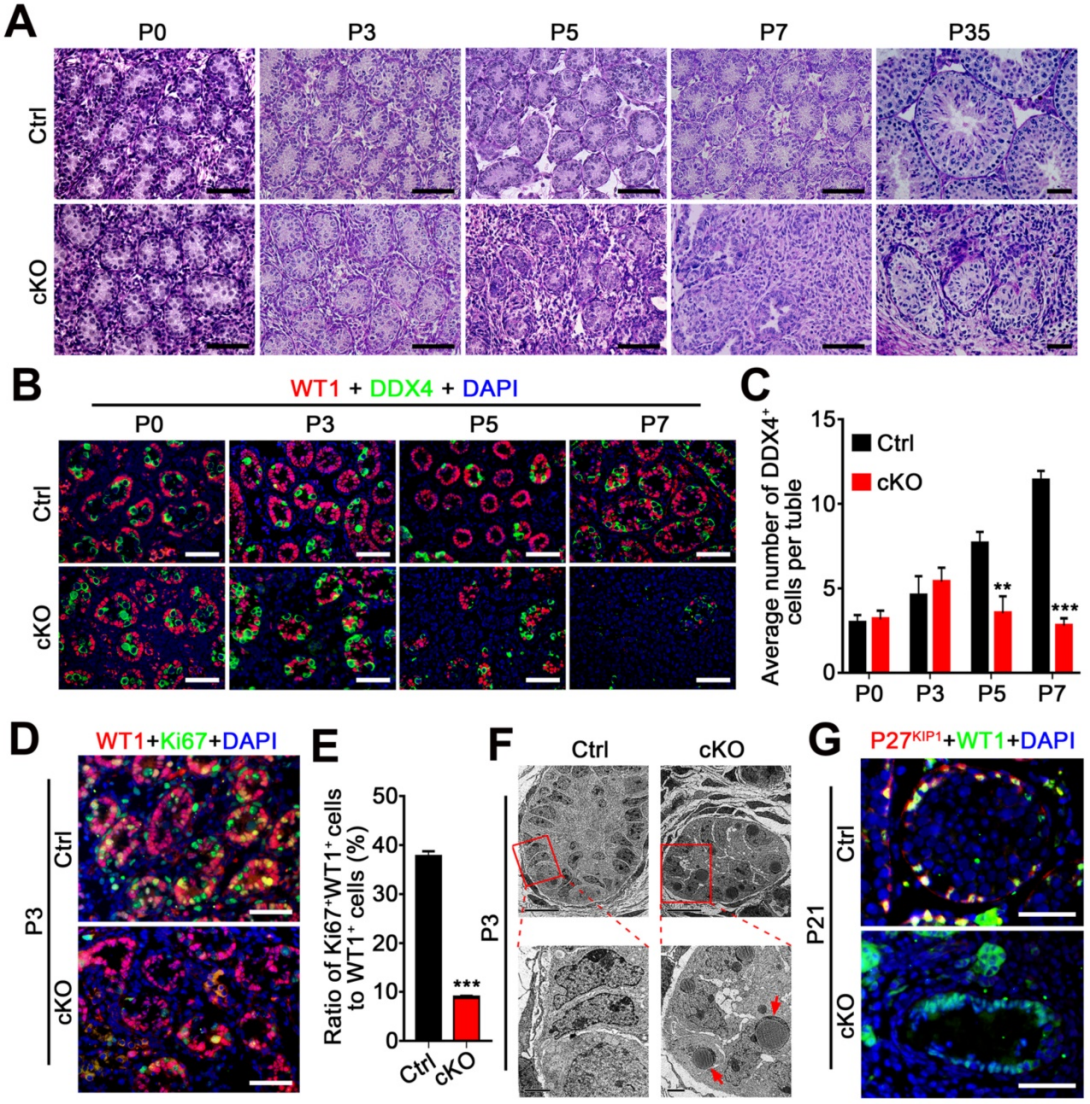
** Loss of hnRNPU in Sertoli cells impairs germ cell and Sertoli cells development and proliferation.** (**A**) Representative PAS-stained paraffin sections of developing testes at P0, P3, P5, P7, and P35 from control and Amh-Cre-cKO mice are shown. Scale bars = 50 µm. (**B**) Co-immunofluorescent staining of WT1 (Sertoli cell marker, red) with DDX4 (germ cell marker, green) in testicular sections at P0, P3, P5, and P7 from control and Amh-Cre-cKO mice are shown. Nuclei were stained with DAPI. Scale bars = 50 µm. (**C**) The histograms showing the quantifications of DDX4^+^ cells per tubule in (B). Data are presented as mean ± SEM, n=3. **P < 0.01 and ***P < 0.001 by Student's* t-*test. (**D**) Co-immunofluorescent staining with WT1 (red) and the Ki67 (green) on testis sections from control and Amh-Cre-cKO mice at P3 are shown. Nuclei were stained with DAPI. Scale bars = 50 µm. (**E**) The histograms showing the quantification of the ratio for Ki67^+^ and WT1^+^ cells to WT1^+^ cells in (D). Data are presented as mean ± SEM, n = 5. ***P < 0.001 by Student's* t-*test. (**F**) Transmission electron microscopy images of control (left panel) and Amh-Cre-cKO (right panel) testis ultra-sections at P3. Lower panels are the zoom-in from the rectangle region of upper panels. Red arrows indicate lipid accumulation. Scale bars = 10 µm in upper panels and 2 µm in lower panels. (**G**) Co-immunofluorescent staining with WT1 (green) and the P27^KIP1^ (red) on control and Amh-Cre-cKO mouse testes at P21 is shown. Nuclei were stained with DAPI. Scale bars = 50 µm.

Although no differences in testis/body weight were observed between control and Amh-Cre-cKO mice at P0-P3, the testis weight was much reduced in Amh-Cre-cKO compared with control males at P5, suggesting severe cell depletion in Amh-Cre-cKO testes at ~P5. Histological analyses confirmed that no significant morphological differences existed from P0 to P3 between Amh-Cre-cKO and control mice (**Figure 3A**). At P5, the seminiferous tubule contents were drastically reduced, and the reduction continued thereafter in Amh-Cre-cKO testes, suggesting severe cell depletion in neonatal Amh-Cre-cKO testes. By P7, many of Sertoli cell-only seminiferous tubules and degenerated tubules were observed in Amh-Cre-cKO testes, whereas well-organized seminiferous tubules containing germ cells and Sertoli cells were present in control testes (**Figure 3A**). In P35 Amh-Cre-cKO testes, seminiferous tubules were rarely seen, and those that remained contained very few degenerated cells (**Figure 3A**). By co-immunostaining with DDX4 (a germ cell marker) and WT1, we examined the number of germ cells in Amh-Cre-cKO and control testes at P0, P3, P5, and P7. Consistent with the histological analyses, no germ cell loss was observed before P3 in Amh-Cre-cKO testes, but the number of germ cells (DDX4 positive) per tubule was decreased markedly at P5 onward (**Figure 3B-C**). These data suggest that the absence of hnRNPU in Sertoli cells affects testicular development and germ cell development after P3.

We next asked whether the Sertoli cell development was affected in Amh-Cre-cKO mice. By analyzing the proliferation capacity of Sertoli cells at P3 testes of control and Amh-Cre-cKO mice, we performed co-immunofluorescence assays using Sertoli cell marker (WT1) and cell proliferation markers (Ki67 and PCNA). The results showed that both the Ki67^+^ Sertoli cells and PCNA^+^ Sertoli cells were significantly decreased in P3 Amh-Cre-cKO testes compared to that of control testes (**Figure 3D-E** and **Figure S2C**), suggesting the pre-pubertal Sertoli cell proliferation was arrested upon hnRNPU deletion in Sertoli cells. In addition, transmission electron microscopy (TEM) analysis revealed that many large, high-density and triangular nuclei of Sertoli cells were located at the base of seminiferous tubules in the control testes at P3 (**Figure 3F**,* left*). In contrast, the nuclei of the Sertoli cell displayed non-dense features and contained multiple lipid droplets in Amh-Cre-cKO testes (**Figure 3F**, *right*). These results suggest that the development of Sertoli cells was disrupted in Amh-Cre-cKO testes. Furthermore, the remaining Sertoli cells (WT1 positive cells) at P21 testes in Amh-Cre-cKO mice do not show any P27^KIP1^ (a Sertoli cell maturation marker 37) positive signals. However, all of the Sertoli cells displayed P27^KIP1^ positive features in control testes at P21 (**Figure 3G**), suggesting the Sertoli cell maturation arrested in Amh-Cre-cKO testes. Together, our data demonstrate that hnRNPU in Sertoli cells is required for pre-pubertal Sertoli cell proliferation, development, and maturation in postnatal testes.

### Loss of hnRNPU in Sertoli cells results in failed spermatogonia development and migration

**Figure 4:**
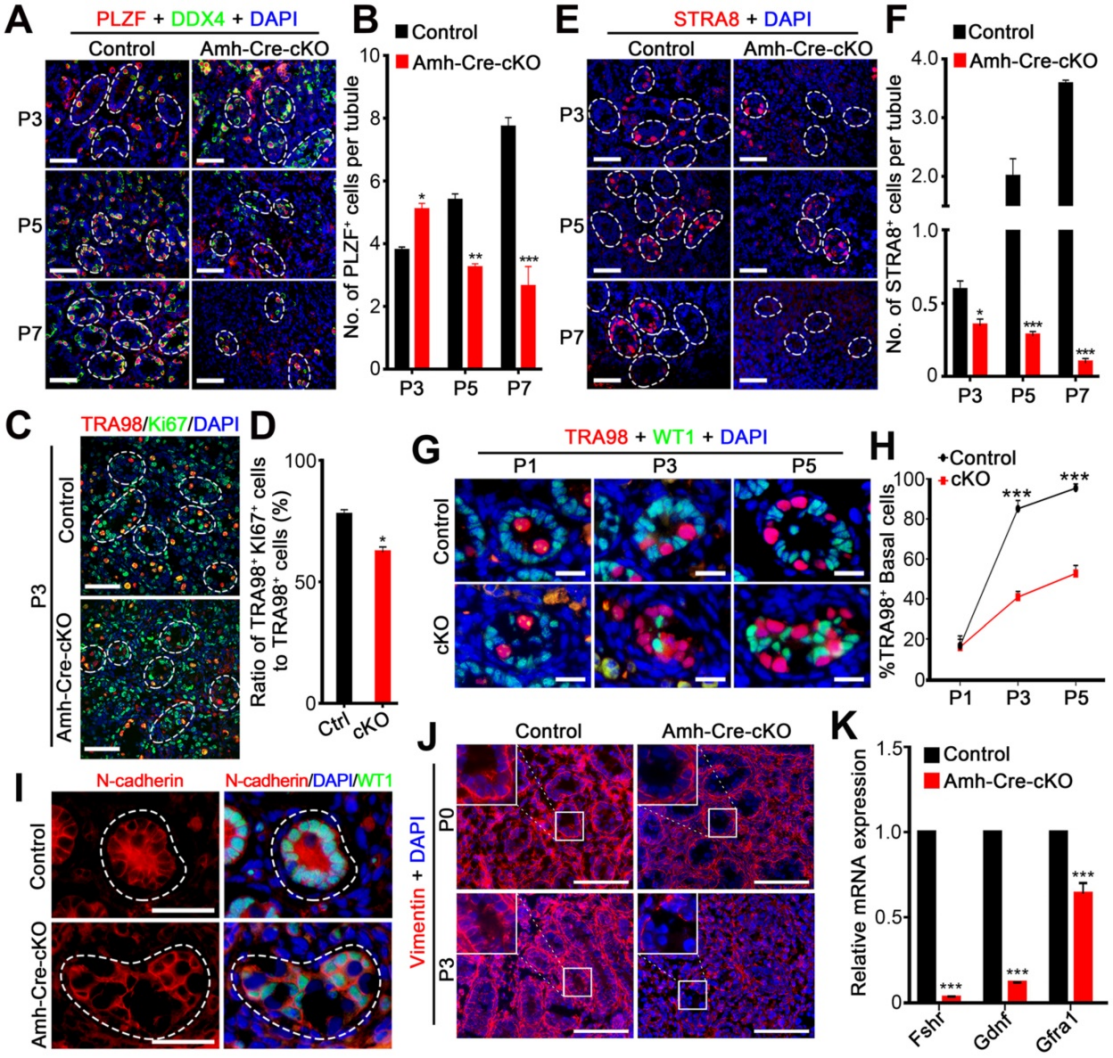
** Ablation of hnRNPU in Sertoli cells causes aberrant spermatogonia development and migration.** (**A**) Co-immunofluorescent staining of PLZF (an undifferentiated spermatogonia marker, red) with DDX4 (green) on testis sections from control and Amh-Cre-cKO mice at P3, P5, and P7 are shown. Nuclei were stained with DAPI. Scale bars = 50 µm. White circles indicate each seminiferous tubules. (**B**) The histograms showing the quantifications of PLZF^+^ cells per tubule in (A). Data are presented as mean ± SEM, n = 3. *P < 0.05, **P < 0.01, ***P < 0.001 by Student's *t*-test. (**C**) Co-immunofluorescent staining of TRA98 (red) with Ki67 (green) in control and Amh-Cre-cKO testis sections at P3 are shown. Nuclei were stained with DAPI. Scale bars = 50 µm. White circles indicate each seminiferous tubules. (**D**) The histograms showing the quantifications of the ratio for TRA98^+^ and Ki67^+^ cells to TRA98^+^ cells at P3 in (C). Data are presented as mean ± SEM, n = 3. *P < 0.05 by Student's *t*-test. (**E**) Immunofluorescent staining of STRA8 (a differentiated spermatogonia marker, red) on testis sections from control and Amh-Cre-cKO mice at P3, P5, and P7 are shown. Nuclei were stained with DAPI. Scale bars = 50 µm. White circles indicate each seminiferous tubules. (**F**) The histograms showing the quantifications of STRA8^+^ cells per tubule in (E). Data are presented as mean ± SEM, n = 3. *P < 0.05, ***P < 0.001 by Student's *t*-test. (**G**) Co-immunofluorescent staining of TRA98 (a germ cell marker, red) with WT1 (a Sertoli cell marker, green) in control and Amh-Cre-cKO testis sections at P3, P5, and P7 are shown. Nuclei were stained with DAPI. Scale bars = 20 µm. (**H**) The quantification of the percentage of the germ cells located at the basal region of the seminiferous tubules in (G). Data are presented as means ± SEM. n = 3. ***P < 0.001 by Student's *t*-test. (**I**) Co-immunostaining of N-cadherin (red) with WT1 (green) on testis sections from control and Amh-Cre-cKO mice at P3 are shown. Nuclei were stained with DAPI. Scale bars = 50 µm. White circles indicate each seminiferous tubules. (**J**) Immunostaining of Vimentin (red) on control and Amh-Cre-cKO mouse testis sections at P0 and P3 are shown. Nuclei were stained with DAPI. Scale bar = 100 µm. (**K**) Histograms showing RT-qPCR analyses of expression levels of *Fshr, Gdnf,* and *Gfrα1* mRNAs in control and Amh-Cre-cKO testes at P3. Data are presented as mean ± SEM, n = 3. ***P < 0.001 by Student's *t*-test.

Given that the number of germ cells in seminiferous tubules was significantly reduced in Amh-Cre-cKO testes after P3 (**Figure 3B-C**), we next asked whether the spermatogonia development was affected in Amh-Cre-cKO testes. To investigate this aim, we performed immunostainings with PLZF (undifferentiated spermatogonia marker) and STRA8 (differentiated spermatogonia marker) in various postnatal testes from control and Amh-Cre-cKO mice. In control mice, the number of PLZF^+^ cells continuously increased from P3 to P7. In contrast, the number of PLZF^+^ cells significantly decreased from P3 to P7 and almost absent in Amh-Cre-cKO testis at P7 (**Figure 4A-B**). This suggests that undifferentiated spermatogonia proliferation was compromised in Amh-Cre-cKO testes. Further co-immunostaining of TRA98 with Ki67 (a proliferation marker) revealed that the number of Ki67^+^/TRA98^+^ cells was significantly decreased in Amh-Cre-cKO testes compared with control testes at P3 (**Figure 4C-D**). Moreover, we observed that the number of STRA8^+^ cells was significantly reduced at P5 and almost disappeared at P7 in Amh-Cre-cKO testes (**Figure 4E-F**). These data indicate that the proliferation and differentiation of spermatogonia in the neonatal mouse testes are affected by the ablation of hnRNPU in Sertoli cells.

In addition, beginning at P3, spermatogonia had already migrated to the basal compartment in control testes; however, the spermatogonia mostly remained in the lumen of the seminiferous tubules in Amh-Cre-cKO testes at P3 and P5 (**Figure 4G-H**), suggesting that Amh-Cre-cKO mouse spermatogonia had lost the ability to locate the basement membrane. We next determined what aspects of germ cell migration were prominently affected by hnRNPU deletion in Sertoli cells. As cell-cell adhesions are critical for cell migration, we examined the distribution patterns of N-cadherin (a Sertoli cell adhesion marker). Unlike the uniform expression of N-cadherin on the cell membrane in control Sertoli cells at P3 testes, a portion of Amh-Cre-cKO Sertoli cells displayed uneven and discontinuous membrane staining of N-cadherin on the cell membrane (**Figure 4I**). Further immunostaining revealed that the Vimentin signals (a cytoplasmic Sertoli cell marker) are much reduced in the Amh-Cre-cKO testes at P3 (**Figure 4J**). Since the primary defects lie in Sertoli cells of Amh-Cre-cKO testes, the failure of spermatogonia development and migration are likely to result from Sertoli cell defects, e.g., a disrupted Sertoli cell niche. Further support this notion is our finding that the transcription levels of *Fshr* (FSH receptor at the surface of Sertoli cells and is a positive regulator of GDNF expression in Sertoli cells) and *Gdnf* (a soluble factor secreted mainly by Sertoli cells and essential for self-renewal and migration of SSCs) 38, 39 are significantly decreased in Amh-Cre-cKO testes at P3 compared with controls (**Figure 4K**). Altogether, our data indicate that hnRNPU-deficient Sertoli cells cannot support normal spermatogonia development and migration.

### hnRNPU enhanced *Sox8* and *Sox9* expression in Sertoli cells by directly binding to their promoter regions

**Figure 5:**
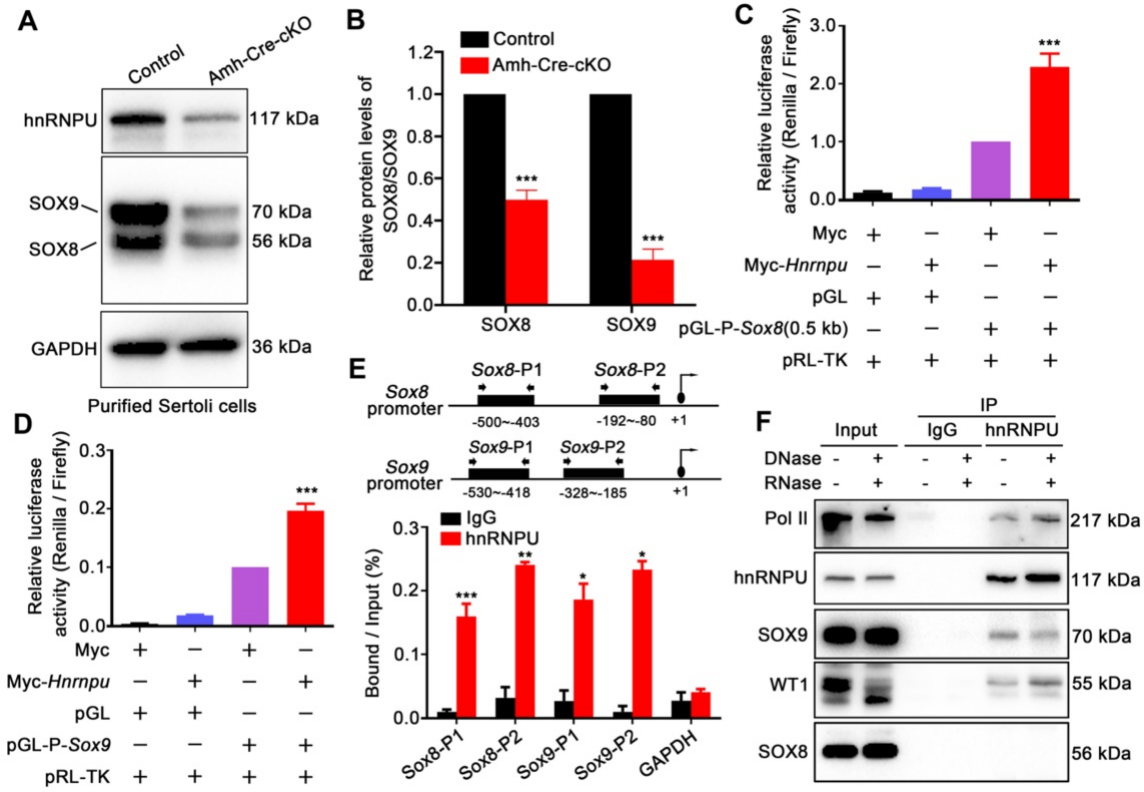
** hnRNPU directly binds to *Sox8/9* promoter regions and regulates its expression levels.** (**A**) Western blot analyses of hnRNPU, SOX8, and SOX9 in Sertoli cells purified from control and Amh-Cre-cKO mouse testes are shown. GAPDH served as a loading control. (**B**) The histograms showing the quantifications of SOX8 and SOX9 protein levels in (A). Data are presented as mean ± SEM, n = 3. ***P < 0.001 by Student's *t*-test. (**C-D**) Luciferase-based reporter assays showing the luciferase activity of the *Sox8* (C) and *Sox9* (D) promoter region in Sertoli cells significantly increase when hnRNPU overexpression. (**E**) ChIP-qPCR shows the hnRNPU enrichments at the different promoter regions of *Sox8* and *Sox9* genes. IgG was used as a negative control for *Sox8/9* binding. The upper schematic image illustrates the position of the forward and reverse primers in two different promoter regions used for the ChIP-qPCR assays. Quantitative data are expressed as the ratio of the ChIP (Bound) to the input DNA. *Gapdh* promoter was used as a negative control for hnRNPU enrichment. *P < 0.05, **P < 0.01, ***P < 0.001 by Student's *t*-test. (**F**) Immunoprecipitation of hnRNPU in isolated Sertoli cells at P3 followed by western blot detection of Pol II, hnRNPU, SOX9, WT1, and SOX8 are shown.

Since transcriptional factor WT1, as a Sertoli cell marker, is essential at multiple steps in testicular development, including sex determination and subsequent development of testes 34, 35, and we found that hnRNPU could interact with WT1 in mouse testis (**Figure 1B**), we sought to ask whether ablation of hnRNPU affects the expression of WT1 target genes, such as *Col4a1* and *Col4a2* 16. As expected, the results showed a significant decrease in mRNA expression levels of *Col4a1* and *Col4a2* in Amh-Cre-cKO testes compared with controls (**Figure S3A**), suggesting that hnRNPU may regulate WT1 target genes by interacting with WT1. In addition, it has been reported that two transcriptional factors, *Sox8* and *Sox9*, exclusively expressed in Sertoli cells, play an essential role in the maintenance of testicular function. Additionally, *Sox8/9* double mutants exhibited a severe testicular development cease and abnormal Sertoli cell development phenotypes 18, which were similar with the Amh-Cre-cKO mice in this study. We then examined the expression of *Col9a3*, *Testatin*,* Occludin*, and *Connexin43*, which are the key genes regulated by *Sox8* and *Sox9* in testes, and the results showed that these target genes were all significantly downregulated in Amh-Cre-cKO testes at P3 compared with that of controls (**Figure S3A**). We next asked if the expression of *Sox8* and *Sox9* was affected in purified Sertoli cells upon hnRNPU deletion. The results revealed that both protein and mRNA expression levels of *Sox8* and *Sox9* appeared to be significantly reduced in Amh-Cre-cKO Sertoli cells compared with controls (**Figure 5A-B** and **Figure S3B**), suggesting hnRNPU could regulate *Sox8/9* expression in Sertoli cells by transcriptional and/or translational enhancement.

To explore how the hnRNPU ablation affects the expression of *Sox8/9* in Sertoli cells at the molecular levels, we constructed a series of luciferase reporter plasmids containing 0.5 Kb, 1 Kb, 1.5 Kb, and 2 Kb fragment upstream of the *Sox8* gene start codon and a *Sox9* gene promoter fragment encompassing nucleotides -553 to -46 bp 40 to examine the regulation of *Sox8/9* expression by hnRNPU. After overexpressing of hnRNPU in primary purified Sertoli cells and HEK293T cells, all four *Sox8* and *Sox9* reporter activities were significantly increased compared to control cells (**Figure 5C-D** and **Figure S3C-D**), indicating that *Sox8/9* expression in Sertoli cells is increased by hnRNPU. To further test whether *Sox8* and *Sox9* is the direct target of hnRNPU, we performed a ChIP-qPCR assay using the anti-hnRNPU antibody in purified Sertoli cells from P3 mice. As shown in **Figure 5E**, two pairs of primers in *Sox8* and/or *Sox9* promoters (*Sox8*-P1, *Sox8*-P2 and/or *Sox9*-P1, *Sox9*-P2) were designed. The ChIP assay results showed that hnRNPU could bind to *Sox8* and *Sox9* promoter regions (**Figure 5E**), suggesting that hnRNPU directs *Sox8* and *Sox9* expression by binding to their promoters. As a chromatin-associated RNA-binding protein, hnRNPU could form complexes with RNA polymerase II to regulate transcriptional processes 41, 42. To further clarify the relationship between hnRNPU and SOX8/9, immunoprecipitation was performed to study the interaction among hnRNPU, SOX8/9, and the Pol II transcription complex. Interestingly, Pol II was identified in the complex binding to hnRNPU, and hnRNPU could interact with SOX9 but not SOX8 in mouse testis with an RNA-independent manner (**Figure 5F**). Together, these results demonstrate that hnRNPU not only directly enhances S*ox8* and *Sox9* expression by binding to its promoter, but also interacts with SOX9 to be involved in regulating the downstream gene expression of *Sox9* in Sertoli cells, thereby controlling Sertoli cell development and male fertility in mice.

### RNA-seq reveals abnormal transcriptome occurs in Amh-Cre-cKO testes at P3

**Figure 6:**
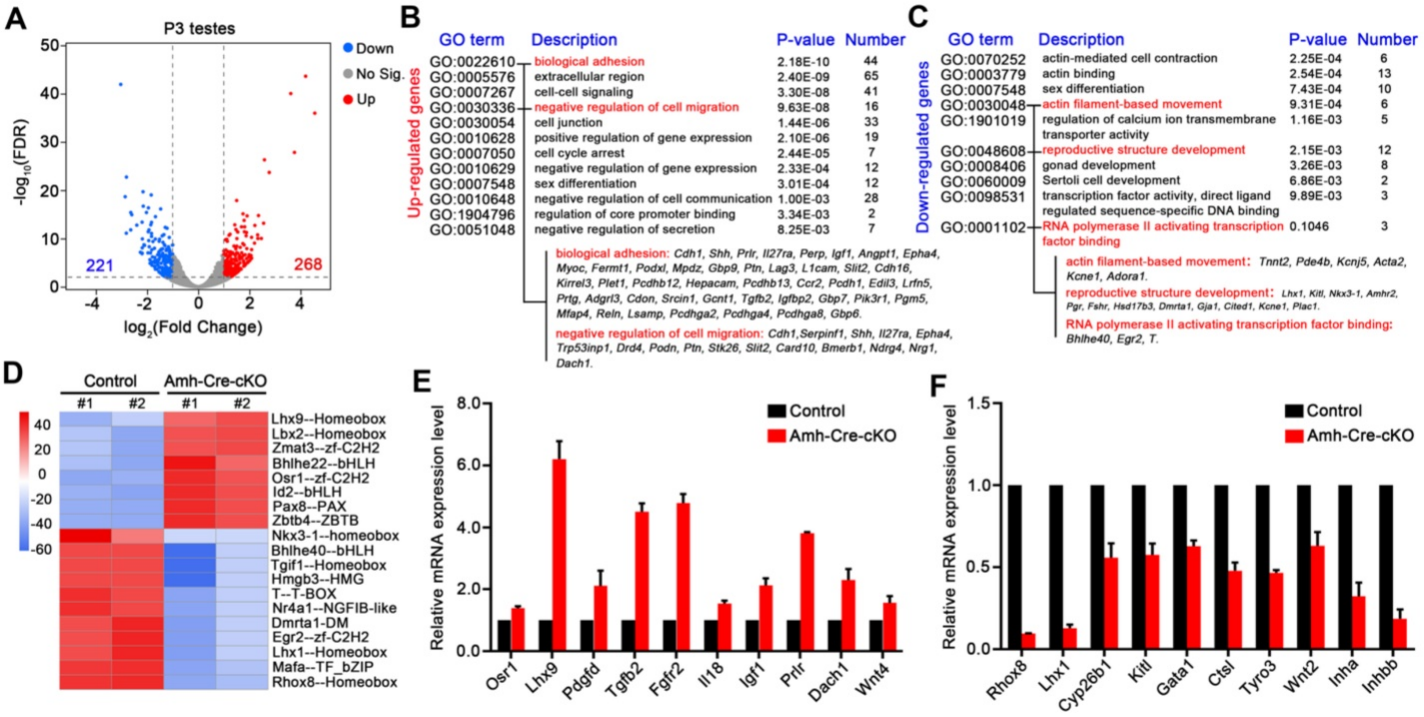
**hnRNPU deficiency in Sertoli cells induces the genome-wide transcriptome profile alterations in testes at P3.** (**A**) Volcano plot showing deregulated genes in Amh-Cre-cKO testes at P3. Significantly regulated genes have an FDR ≤ 0.01 and fold change ≥ 2. (**B-C**) Gene ontology term analyses of the 268 upregulated genes (B) and 221 downregulated genes (C) in Amh-Cre-cKO testes at P3 are shown. The 12 enriched GO pathways in the upregulated genes and 10 enriched GO pathways in the downregulated genes are illustrated by gene counts and *P-*values. (**D**) Heat-map shows the differences in transcriptional activity of transcription factors between control and Amh-Cre-cKO testes analyzed by CoRegNet analysis. (**E-F**) RT-qPCR validates the selected upregulated (E) and downregulated (F) genes in Amh-Cre-cKO testes from RNA-seq data. Data are presented as mean ± SEM, n = 3.

To understand the impact and molecular changes of the loss of hnRNPU in Sertoli cells, we next analyzed transcriptional profiling through RNA sequencing (RNA-seq). Due to the histology and cell population similarity at P0 and P3 testes in Amh-Cre-cKO and control mice, we chose both P0 and P3 testes to perform RNA-Seq. Hierarchical clustering of differentially expressed genes (DEG) showed that in P0, only 11 genes were upregulated and 39 genes were downregulated even when using a low parameter criterion (FDR ≤0.05, fold change ≥1.5) in Amh-Cre-cKO mice compared with control mice (**Figure S4A-B** and **Table S2**). These results suggest that the transcriptome changes were minimal in Amh-Cre-cKO testes at P0, and the molecular effects may not appear at P0 when hnRNPU was absent in Sertoli cells. We then analyzed the transcriptome at P3 testes and found that 489 genes were deregulated in Amh-Cre-cKO mice compared with control mice using a common parameter criterion (FDR ≤ 0.01, fold change ≥2), among which 268 genes were upregulated and 221 genes were downregulated (**Figure 6A** and **Table S3**). Further gene ontology (GO) term enrichment analyses revealed that the upregulated transcripts were mainly enriched in terms related to cell migration, adhesion and communication, such as “biological adhesion” and “negative regulation of cell migration”. On the other hand, the downregulated transcripts were mostly involved in terms related to cell movement, development, and regulation of transcription, such as “actin filament-based movement”, “reproductive structure development” and “RNA polymerase II activating transcription factor binding”, which are consistent with the phenotypic and functional results of Amh-Cre-cKO mice (**Figure 6B-C** and **Table S4-5**).

Interestingly, for the downregulated genes, we found *Lhx1*, *Kitl, Pgr*, and *Fshr* were enriched in terms of “reproductive structure development”, suggesting that it may be involved in regulating testis development. In addition, gene set enrichment analysis (GSEA) showed that the deregulated transcripts were significantly enriched for those that function in cell proliferation, cell junction and adhesion **(Figure S4C)**, which further indicates the transcriptome changes in Amh-Cre-cKO testes were the molecular cause of male infertile phenotype. Therefore, these bioinformatics analyses imply that hnRNPU in Sertoli cells may participate in the gene network of cell proliferation/migration, cell junction and adhesion and regulate gene transcriptional process, thereby contributing to the formation of seminiferous tubule structure and development of Sertoli cells.

As hnRNPU is an important transcription co-regulator, we next evaluated the activities of transcription factors from the RNA-seq data using the CoRegNet analysis method 43. The results advised that there were 19 distinctly differential transcription factors between Amh-Cre-cKO and control testes at P3 **(Figure 6D)**. We then chose 3 transcription factors associated with Sertoli cell and testis development, including *Rhox8* 44, *Lhx1* 45, and *Osr1* 46, to validate the RNA-seq data. The RT-qPCR results were in keeping with the RNA-seq data **(Figure 6E-F)**. Next, we selected several differentially expressed genes which are related to spermatogonia migration (*Kitl* and *Pdgfd*) 22, 23 and which are target genes of *Sox9* (*Cyp26b1, Gata1* and* Ctsl,* etc.) 47 for further confirmation. Indeed, RT-qPCR analyses revealed that the expression levels of these genes were significantly deregulated in Amh-Cre-cKO testes at P3 compared with that of controls **(Figure 6E-F)**. Furthermore, we validated three WT1 target genes, *Inha*, *Wnt4* and *Igf1* 48, were significantly deregulated in Amh-Cre-cKO testes by RT-qPCR, suggesting that interaction of hnRNPU with WT1 may regulate the downstream target genes of WT1 in testes as well. Altogether, the above analyses reveal that hnRNPU in Sertoli cells plays a critical role in testicular development by preventing ectopic expression of genes associated with Sertoli cell and germ cell development.

## Discussion

During neonatal development of mouse testis, the microenvironments built by Sertoli cell-derived regulatory factors support the spermatogonia from the middle of the testis cord to the basement membrane. Although several Sertoli cell-derived KITL, PDGF, and CXCL12 may be involved in maintaining the microenvironments to regulate the migration of germ cells 22, 23, 49, the role of Sertoli cells in gonocyte proliferation and migration processes remains largely unknown. In the current study, we have demonstrated for the first time* in vivo*, using a conditional *Hnrnpu* knockout mouse strain, that hnRNPU plays a central role in regulating Sertoli cell function and neonatal testicular development, and that deactivation of hnRNPU in Sertoli cells leads to severe testicular atrophy and male infertility. Further phenotypic analyses found that loss of hnRNPU in Sertoli cells disrupted its development and resulted in aberrant spermatogonia development and migration during the neonatal testis development. These results suggest that hnRNPU is one of the core regulatory factors which modulates neonatal Sertoli cell and testis development. Mechanism studies showed that hnRNPU could directly enhance *Sox8* and *Sox9* expression through binding to its promoter region. On the other hand, hnRNPU also interacts with WT1 and SOX9 and regulates the expression of Sertoli cell-derived genes, such as C*yp26b1*, that are related to neonatal testis development.

It is well known that WT1, as one of the Sertoli cell markers, is a crucial regulator of Sertoli cell function and testes development and plays an essential role in male fertility of humans and mice 12, 16, 34, 35. Loss of function of WT1 in Sertoli cells (*Amh-Cre* induced) caused male infertility phenotype with testicular cords disruption, and basal lamina fragmentation occurred as early as at E15.5 testes 34, which is much earlier than Amh-Cre-cKO males in this study. Besides, we found that hnRNPU could interact with WT1 in mouse Sertoli cells, suggesting hnRNPU may regulate Sertoli cell function by cooperating with WT1. Interestingly, hnRNPU has been reported to interact with WT1 to modulate WT1 transcriptional activation in embryonic stem (ES) cells 33, raising a possible typical role of hnRNPU in different mammalian cell types by regulating *Wt1* target genes.

In addition, we found that Amh-Cre-cKO testes display no significant differences to control at histological levels at P0, but the migration and development of spermatogonia exhibited abnormities at P3, indicating that Sertoli cell development and niche formation were destroyed at P3. Indeed, we found abnormalities in the cytoskeleton and adhesion of Amh-Cre-cKO Sertoli cells at P3. Transcriptome data of P3 testes also showed that deregulated genes in Amh-Cre-cKO testes were related to cell migration, biological adhesion, and reproductive structure development, which could explain, to some extent, the phenotypic characteristics of Amh-Cre-cKO mice on molecular levels. Inspiringly, based on RNA-seq data, we found some SOX9 and WT1 target genes were deregulated in Amh-Cre-cKO testes, which might also be essential for causing the defects of Sertoli cell and testicular development. For instance, a decrease of *Cyp26b1*, a SOX9 target gene, upon hnRNPU depletion in this study may result in the increase of RA and Sertoli cells enter the differentiated stage prematurely, leading to the loss of SSCs pool because *Cyp26b1* could metabolize and inactivate retinoic acid (RA) in Sertoli cells, and maintain male germ cells in the undifferentiated state 50. We also found SOX9 target gene *Gata1*, a transcriptional factor of FSHR 51, is downregulated in hnRNPU-deficient testes, and the FSH pathway was reported to regulate RA signaling and GDNF expression to commit spermatogonial self-renewal 52, 53. In addition, *Igf1* and *Wnt4*, two potential target genes of WT1, were considered to play an essential regulatory role in sex determination and maintenance, and the insulin/IGF or WNT4 signaling pathway is also required for FSH-mediated SC proliferation and testicular development 54-56. Importantly, in the current study, we also found both *Igf1* and *Wnt4* were upregulated in hnRNPU-deficient testes at P3. Therefore, as a critical regulatory factor in Sertoli cells, hnRNPU may play a central role in regulating upstream signal reception and control the secretion of related regulatory factors for germ cell proliferation and migration by directly modulating *Sox8/9* or interacting with WT1 further to regulate their target genes at the transcriptome levels. It is worth noting that RT-qPCR shows a significant decrease of *Sox8/9* expression levels in hnRNPU-deficient Sertoli cells, but the RNA-seq data did not detect the downregulated *Sox8/9* in hnRNPU mutant testes. This discrepancy is usually caused by RNA-Seq data processing and analysis limitations because it is challenging to detect the differential genes that belong to a larger gene family and have fewer exons 57, 58. *Sox8/9* are members of a large Sry-related HMG BOX (SOX) gene family, and both of them only have three exons, which may explain why the expression of *Sox8/9* was not observed down-regulated in the RNA-Seq data. Notably, our qPCR and Western blot data are all showing a significant decrease in *Sox8/9* expression in hnRNPU mutant testes, indicating that qPCR is still considered a reliable method of choice to validate RNA-Seq data.

Interestingly, although the Sertoli cells in Amh-Cre-cKO mice at P3 displayed no discernible differences compared to that of controls at histological levels, the proliferation capacity of Sertoli cells was significantly decreased. Moreover, we also observed that spermatogonia development was affected in Amh-Cre-cKO testes at P3, including spermatogonia migration. These results raised a possibility that the molecular changes in Amh-Cre-cKO testes occurred prior to the time point when the histological phenotype just appeared at P3 testes. Interestingly, our RNA-seq analyses of Amh-Cre-cKO testes at P0 revealed only 50 deregulated genes using a low parameter, but there are also 25 deregulated genes consistent with the Amh-Cre-cKO testes at P3. Therefore, the earliest possible time point for the abnormalities in Amh-Cre-cKO is the preparatory phase of prospermatogonial transformation after birth. The onset of abnormal spermatogonia development and migration at P3 in Amh-Cre-cKO testes coincides with the transition time of Prospg^T1^ to Prospg^T2^ and the establishment of spermatogonial stem cells' (SSCs) niche 10, 59. Thus, our findings support the notion that hnRNPU in Sertoli cells plays a crucial role in neonatal Sertoli cell development and niche maintenance for the transition of spermatogonia and establishment of SSCs.

As a co-transcription factor, hnRNPU could interact with the RNA polymerase II complex and related transcription factors to regulate the synthesis of transcription processes 42, 60, 61. In addition, hnRNPU interacts with several long noncoding RNA to regulate senescence entry and exit, inflammatory gene expression, brown and beige fat development, and X-chromosome inactivation 62-66. Recent studies showed that active gene promoters are major hotspots for interaction with RNA-binding proteins, and most of the hnRNP family proteins are directly involved in transcription control 67, 68. In this study, we found that hnRNPU could directly bind to the promoter region of *Sox8* and *Sox9* and enhance their expression, but ablation of hnRNPU in Sertoli cells didn't completely suppress *Sox8* and *Sox9* expression. Interestingly, our Sertoli cell-specific hnRNPU mutant testes showed similar phenotypes with *Sox8/9* double nullizygous testes from P3, whereas *Sox8/9* double nullizygous testes showed an earlier phenotype in embryonic day 18. This may be caused by the spatiotemporal regulation of hnRNPU during testicular development from embryonic day to postnatal day. Although hnRNPU is expressed in Sertoli cells at the embryonic stage, its main regulatory function might be in the postnatal stage. It is worth noting that in this study, hnRNPU was found to be highly expressed in most types of male germ cells as well, including prospermatogonia, spermatogonia, spermatocytes, and round spermatids, indicating that hnRNPU also plays an important physiological role in the development of male germ cells. However, this needs further research in the future.

In summary, our current study identified an important role of hnRNPU in Sertoli cell development and establishing a Sertoli cell niche for pro-spermatogonia development and migration in neonatal mouse testes. Our findings provide a novel understanding of the regulatory network of hnRNPU in Sertoli cells that regulates testicular development by enhancing the expression of core transcription factors *Sox8* and *Sox9* and regulating their target genes in Sertoli cells. Furthermore, our study also suggests that hnRNPU affect the expression of many transcription factors associated with Sertoli cell and testis development at the whole transcriptome level, such as *Rhox8*, *Lhx1*, and *Osr1*, et al., which further ensures the normal development of Sertoli cells and the establishment of spermatogenic tubule structures.

## Materials and methods

### Animals

All animal work was performed and approved by the Institutional Animal Care and Use Committee (IACUC) of Tongji Medical College, Huazhong University of Science and Technology. All mice were housed in the specific pathogen-free animal facility of Huazhong University of Science and Technology. Floxed* Hnrnpu* transgenic mice (*Hnrnpu^flox/flox^*) were a gift from Dr. Tom Maniatis's lab at Columbia University. The *Amh-Cre* deletor mice were purchased from Jackson Laboratory and maintained on the C57BL/6J background. To obtain *Amh-Cre; Hnrnpu^flox/flox^* (designated as Amh-Cre-cKO) male mice, Amh-Cre males were mated with *Hnrnpu^flox/flox^* females initially to generate the Amh-Cre;* Hnrnpu^+/flox^*. Then they were bred with *Hnrnpu^flox/flox^* mice to generate Amh-Cre-cKO males. The genotyping strategy of *Hnrnpu*-floxed mice was performed as the previous report 31.

### Human testis sample collection

Human fetal testis samples were obtained from aborted fetuses in inevitable abortion, such as premature rupture of membranes. Adult testis samples were collected from the remaining pieces after microdissection of testicular sperm extraction (micro-TESE) for obstructive azoospermia patients. All procedures were performed following the protocols approved by the Medical Ethics Committee of the Reproductive Medicine Center of Tongji Medical College of Huazhong University of Science and Technology. All participating patients have signed the informed written consent to the research process.

### Mouse fertility evaluation

Adult control and Amh-Cre-cKO males (n = 6 for each genotype) were bred and monitored for 12 weeks. One male mouse was housed with two adult WT fertility-proven females for at least five months. Cages were monitored daily, and pup numbers and litters were recorded.

### Histology and immunofluorescence

Mouse testes and epididymides of different ages were dissected and fixed in Bouin's solution (Sigma, HT10132) at 4 °C overnight and then stored in 70% ethanol. Samples were dehydrated for paraffin embedding. 5 μm sections were cut and stained with hematoxylin and eosin after being dewaxed and rehydrated. For immunofluorescence, testes were fixed in 4% paraformaldehyde overnight at 4 °C and then were dealt with gradient sucrose and embedded in O.C.T. (Sakura Finetek, 4583) medium (50% OCT plus 10% sucrose). 5 μm cryo-sections were cut and subjected to antigen retrieval in a microwave oven with 0.01 M sodium citrate buffer (pH = 6.0). The nonspecific activity was blocked in blocking solution (containing 5% normal donkey serum and 5% fetal bovine serum in 1% bovine serum albumin) for 1 h at RT. Subsequently, sections were incubated overnight at 4 °C with primary antibodies (**Table S6**) diluted in blocking solution. After washing, slides were incubated with secondary antibody (**Table S6**) in the dark for 1 h at RT and Vectashield mounting medium containing DAPI (H1200, Vector laboratories) was applied to the samples. Sections were captured using a FluoView 1000 microscope (Olympus, Japan) with digital camera (MSX2, Micro-shot Technology Limited, China).

### Western blotting

About 15-20 μg of protein lysates from testes or Sertoli cells (lysed in RIPA buffer (CWBIO, Cat# 01408) plus 1× proteinase inhibitor) were subjected to SDS/PAGE gel for Western blot analysis. After 1-2 h of electrophoresis, the protein was transferred to the PVDF membrane (Bio-Rad) under a 280 mA constant current for 90 min. Then, the PVDF membrane was put into the plastic bowl containing 5% non-fat milk and shaken slowly for 1 h at RT. The primary antibody diluted with 5% non-fat milk was incubated with the membrane at 4 °C overnight. Then the membrane was washed in TBST three times for 10 min and incubated with a secondary antibody immediately at RT for 1 h. After using Luminol/enhancer solution and Peroxide solution (ClarityTM Western ECL Substrate, Bio-Rad), the membrane was exported by a chemiluminescence imaging system (Gel Doc XR+ System, Bio-Rad) to detect the objective bands.

### RNA isolation and quantitative RT-PCR

Total RNAs were isolated from testes or Sertoli cells with TRIzol reagent (Invitrogen), and the RNA concentration was measured by a Nanodrop ND-2000 spectrophotometer (Thermo Scientific). Total RNA (0.5-1 µg) was reverse transcribed to cDNA using a Hiscript^®^Ⅱ 1st Strand cDNA Synthesis Kit (Vazyme) according to the protocol. The reaction system was 20 µl, including 10 μl 2 × RT mix, 2 μl HiScriptⅡ Enzyme Mix, 1 μl random hexamers (50 ng/μl), 1 μl Oligo(dT)_23_VN (50 μM), 4 μl RNase free ddH_2_O, and 2 μl template RNA. The reaction conditions were 25 °C, 5 min; 55 °C, 15 min; 85 °C, 2 min; 4 °C, 5 min. The obtained cDNA was diluted to 100 ng/µl and applied for RT-qPCR analysis, which was performed in Step One ABI real-time PCR System through SYBR^®^ green master mix (YEASEN). The RT-qPCR conditions were as follows: 95 °C for 5 min, followed by 40 cycles of 95 °C for 10 s, 60 °C for 30 s, 95 °C for 15 s, 60 °C for 1 min, and 95 °C for 15 s. Each reaction consisted of 10 µl of SYBR Green, 1 µl of 100 µM sense primer, 1 µl of antisense primer, 2 µl of cDNA, and 6 µl of H_2_O, for a total volume of 20 ul. The primers used for RT-qPCR are listed in **Table S7.**

### Isolation of Sertoli cells

The mouse testes at P3 were dissected and washed with DHANKS medium and transferred to the clean bench to strip the tunica albuginea. Then the testicular tissues were immediately transferred into a 15 ml sterile centrifuge tube containing 10 ml DMEM/F12 medium comprised 1 mg/mL collagenase IV (Sigma, Cat. No. C5138-100 mg), 0.5 mg/mL Deoxyribonuclease I (Sigma, Cat. No. DN25-100 MG) and 0.5 mg/mL hyaluronidase (Sigma, Cat. No. H3506-100 MG). After digestion at 37 °C on a shaker for 15 min, the seminiferous tubules were allowed to settle and were subsequently washed twice with 2-3 ml DMEM/F12 medium. Then 2.5 mg/ml trypsin and 0.5 mg/mL Deoxyribonuclease I were added to the solution at 37 °C on a shaker for 15 min for further digestion. Afterward, the digestion was stopped by adding DMEM/F12 containing 10% FBS, and the suspension was filtered with 40 µm pore-size nylon mesh. The filtered solution was collected in a 15 ml sterile centrifuge tube and centrifuged at 1000 rpm/min for 5 min. The Sertoli cells were collected after washing once with 2-3 ml DMEM/F12 medium for 1 min. Then the Sertoli cells were resuspended in DMEM/F12 containing 10% fetal calf serum, 100 U/ml of penicillin, and 100 μg/ml of streptomycin sulfate and cultured in a 60 mm dish at 35 °C and 5% CO_2_. After 48 h of culture, Sertoli cells had grown on the wall and showed irregular polygonal shape, and then the Sertoli cells were collected and prepared for the follow-up experiments.

### Transmission electron microscopy (TEM)

TEM was performed as described previously with minor modifications 69. Briefly, the testes collected from Amh-Cre-cKO and control mice at P3 were fixed with electron microscope fixative solution (0.1 M cacodylate buffer (pH = 7.4) containing 3% paraformaldehyde and 3% glutaraldehyde plus 0.2% picric acid) for 2 h in 4 °C, then for 1 h at RT. Testes were fixed again for 1 h with 1% osmium acid at RT after being washed by 0.1 M dimethylarsenate buffer solution 3 times, each time for 10 min.Then the testes were treated with gradient alcohol dehydration and embedded with an Eponate mixture (Electron Microscopy Sciences, Hatfield, PA, USA) at 60 °C overnight. The samples were cut into 60-70 μm thick sections with diamond knives using an ultrathin microtome. The ultra-sections were stained with 3% uranium acetate and lead citrate, then observed with a transmission electron microscope (CM120 BioTwin Philips) and photographed.

### TUNEL staining

Apoptotic germ cells were detected by TUNEL staining using I Colorimetric TUNEL Apoptosis Assay Kit (Beyotime) following the manufacturer's instructions. Briefly, the paraffin sections of Amh-Cre-cKO and control testes were placed in a glass tank filled with xylene for 5 min to dewax. This step was repeated three times. Followed by hydration in gradient alcohol, and then incubated with Tris-HCl (0.1 M, pH = 7.5) containing 3% BSA and 20% fetal bovine serum for 30 min. After incubation, the samples were washed three times in PBS (5 min per wash), then covered with the TUNEL reaction mixture in a wet box at 37 °C for 1 h. After being washed three times in PBS, DAPI was used to stain the nuclei. The signals were observed under a FluoView 1000 microscope (Olympus, Japan).

### Immunoprecipitation

For immunoprecipitation, cells or testicular tissue were homogenized in iced lysis buffer (20 mM HEPES, 150 mM NaCl, 2 mM magnesium acetate, 0.2% NP-40, 1 mM DTT, pH = 7.3), shake at 4 °C on a rotator for 30-60min, then centrifugated at 12000 rpm, 4 °C for 15 min. 50 μl protein supernatant was taken as input and the remaining protein supernatant was used to incubate with antibody-coupled Protein A beads or Protein G beads (Bio-Rad, 161-4013) according to the manufacturer's Instructions at 4 °C overnight. The next day, the protein-antibody-bead complex was washed with lysis buffer 5-6 times. After washing, the proteins were eluted from the beads with a 2 x SDS loading buffer and subjected to western blotting procedures.

### Plasmid constructs and transfections

The cDNA of *Hnrnpu* was amplified from mouse testis RNA and subcloned into pCMV-MYC vector using ClonExpress II One Step Cloning Kit (Vazyme Biotech, Nanjing, China), which is named MYC-*Hnrnpu*. The promoter region of *Sox8* and *Sox9* were amplified and subcloned in pGL4.10 vector and named pGL-P-*Sox8/9*. The primers are listed in **Table S7**. Transfections were performed with Lipofectamine 2000 (Invitrogen) for HEK293T cells or Lipofectamine LTX for cultured Sertoli cells according to the manufacturer's instructions. All DNA plasmids were cleared of endotoxin. After 48 h, the transfected cells were collected for further experiments.

### Luciferase reporter assay

Purified Sertoli cells or HEK293 cells were transfected with *Sox8* and *Sox9* promoter into luciferase reporter plasmids (120 ng) using Lipofectamine LTX transfection reagent when cells were ~80% confluent. Cells were harvested and lysed 36 h later. Luciferase activity was measured using a dual-luciferase reporter assay system following the manufacturer's instruction (Promega).

### ChIP-qPCR (chromatin immunoprecipitation quantitative PCR)

ChIP was carried out according to the ChIP Protocol optimized for RNA-binding proteins as previously 68. Briefly, magnetic beads were blocked with glycogen, BSA and tRNA in ChIP dilution buffer. Then magnetic beads were coupled with anti-hnRNPU antibodies. About 1 × 10^7^ Sertoli cells were crosslinked in 1% formaldehyde diluted in PBS for 20 min and then quenched with glycine. Suspension Sertoli cells were resuspended in cell lysis buffer. Nuclei were incubated on ice in nuclear lysis buffer and sonicated with a Sonifier cell disruptor (JY92-IIN, Scientz) to break DNA to a length of approximately 100-600 bp. Immunoprecipitation was performed with hnRNPU antibody and IgG coupled beads and 2% of nuclear lysate was saved. After de-crosslink, input and immunoprecipitated chromatin DNAs were recovered by RNase A digestion, proteinase K treatment, phenol/chloroform extraction and precipitation with ethanol. Immunoprecipitated and input DNAs were analyzed by real-time SYBR Green qPCR (Quantagene q225, KUBO). The primers are listed in **Table S7**.

### RNA-Seq analysis

Total RNA was isolated from P0 and P3 mouse testes using TRIzol reagents (Thermo Fisher Scientific). The RNA concentration was verified using a NanoDrop 2000 Spectrophotometer (Thermo Fisher Scientific). One microgram of total RNA was used from each sample to prepare the mRNA libraries using TruSeq Stranded mRNA Library Preparation Kit Set A (Cat. No. RS-122-2101, Illumina) according to the manufacturers' instructions. All libraries were sequenced using the Illumina HiSeq 4000 platform. The FASTX-Toolkit was used to remove adaptor sequences and low-quality reads from the sequencing data. To identify all the transcripts, we used Tophat2 (v2.0.13) and Cufflinks (v2.1.1) to assemble the sequencing reads based on the UCSC MM10 mouse genome. The differential expression analysis was performed by Cuffdiff (v2.1.1). The differential expressed genes were set with the threshold of FDR ≤0.01, fold change ≥2 for P3 and with the threshold of FDR ≤0.05, fold change ≥1.5 for P0.

### Statistical analysis

All data are presented as mean ± SEM unless otherwise noted in the figure legends. Statistical differences between datasets were assessed by one-way ANOVA or Student's *t*-test using the SPSS16.0 software. P-values are denoted in Figures by *P < 0.05; **P < 0.01; and ***P < 0.001.

## Supplementary Material

Supplementary figures.

Supplementary table 1.

Supplementary table 2.

Supplementary table 3.

Supplementary table 4.

Supplementary table 5.

Supplementary table 6.

Supplementary table 7.
