# Supplementary material for: hnRNPU in Sertoli cells cooperates with WT1 and is essential for testicular development by modulating transcriptional factors Sox8/9
Source: Theranostics. 2021 Oct 25;11(20):10030–46. doi: 10.7150/thno.66819 (PMC8581416; doi:10.7150/thno.66819)
Supplement: Supplementary file 1 — Supplementary figures. [file thnov11p10030s1.pdf]

Supplementary Materials for

hnRNPU in Sertoli cells cooperates with WT1 and is essential for testicular development by modulating transcriptional factors *Sox8/9*

Yujiao Wen, Xixiang Ma, Xiaoli Wang, Fengli Wang, Juan Dong, Yanqing Wu, Chunyu lv, Kuan Liu, Yan Zhang, Zhibing Zhang, Shuiqiao Yuan

***Corresponding author Email:** shuiqiaoyuan@hust.edu.cn

This PDF file includes:

Figure S1. Expression profiles of hnRNPU in multiple tissues and developing testes in mice.

Figure S2. Loss of hnRNPU in Sertoli cells causes apoptosis and proliferation abnormalities in neonatal testes.

Figure S3. hnRNPU deficiency in Sertoli cells affects the gene transcription levels in Sertoli cells.

Figure S4. Loss of hnRNPU in Sertoli cells slightly alters genome-wide transcriptome profiles at P0 testes.

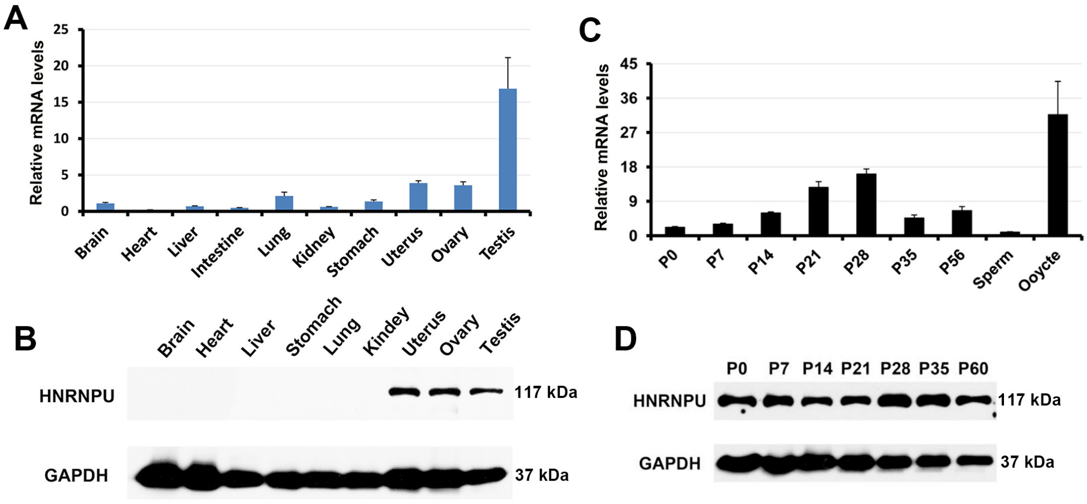

E

	H. sapiens	*M. mulatta*	*B. taurus*	*C.l. familiaris*	*R. norvegicus*	*M. musculus*	*G. gallus*	*X. tropicalis*	*D. rerio*
H. sapiens	100	99.64	98.55	98.72	96.87	97.25	86.96	75.00	61.06
M. mulatta	99.64	100	98.42	98.44	96.62	96.87	86.72	74.87	60.92
B. taurus	98.55	98.42	100	98.86	97.24	97.62	86.96	74.74	61.20
C.l. familiaris	98.72	98.44	98.86	100	97.86	98.29	89.96	77.12	62.84
R. norvegicus	96.87	96.62	97.24	97.86	100	99.62	86.80	75.07	61.70
M. musculus	97.25	96.87	97.62	98.29	99.62	100	86.96	75.07	61.70
G. gallus	86.96	86.72	86.96	89.96	86.80	86.96	100	72.61	61.36
X. tropicalis	75.00	74.87	74.74	77.12	75.07	75.07	72.61	100	59.07
D. rerio	61.06	60.92	61.20	62.84	61.70	61.70	61.36	59.07	100

Figure S1. Expression profiles of hnRNPU in multiple tissues and developing testes in mice. (A) RT-qPCR analyses of the Hnrnpu mRNA levels in 10 mouse tissues. Data are presented as mean ± SEM, n = 3. (B) Western blot analyses of the expression of hnRNPU protein in nine mouse organs are shown. Levels of hnRNPU in multiple organs were determined using western blot analyses. GAPDH was used as a loading control. (C) RT-qPCR analyses of Hnrnpu mRNA levels in developing testes at postnatal day 0 (P0), P7, P14, P21, P28, P35, P56, and in sperm and oocyte. Data are presented as mean ± SEM, n = 3. (D) Western blot analyses of the expression of hnRNPU protein in developing testes are shown. GAPDH served as a loading control. (E) A high degree of conservation of hnRNPU in amino acid sequences among 9 species.

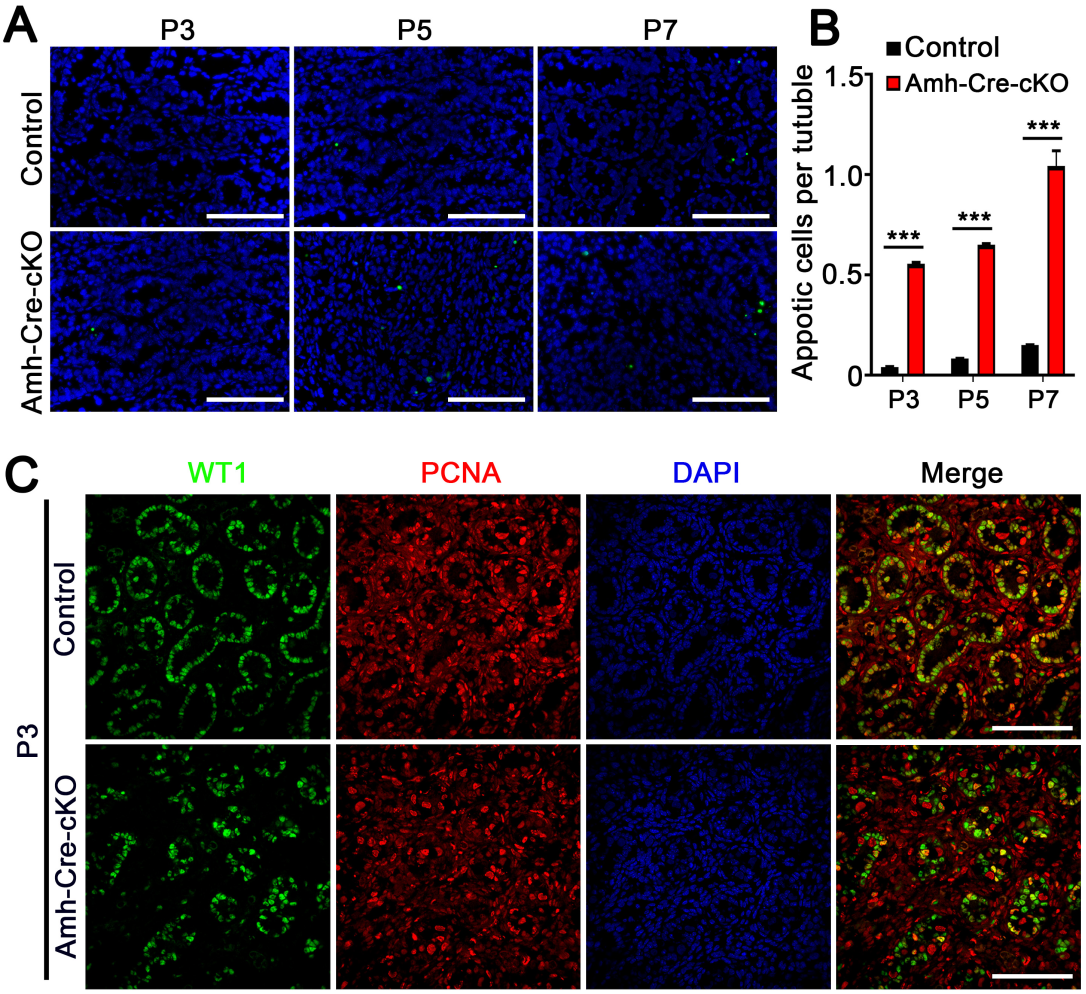

Figure S2. Loss of hnRNPU in Sertoli cells causes apoptosis and proliferation abnormalities in neonatal testes. (A) Representative images of TUNEL assays on testis sections from control and Amh-Cre-cKO mice at P3, P5, and P7 are shown. Scale bars = 100 μm. (B) Histogram shows the quantification of apoptotic cells in (A). Data are presented as mean ± SEM, n = 3. ****P* < 0.001 by Student's t-test. (C) Co-immunostaining of PCNA (red) with WT1 (green) on testis sections from control and Amh-Cre-cKO mice at P3. Nuclei were stained with DAPI. Scale bars = 100 μm.

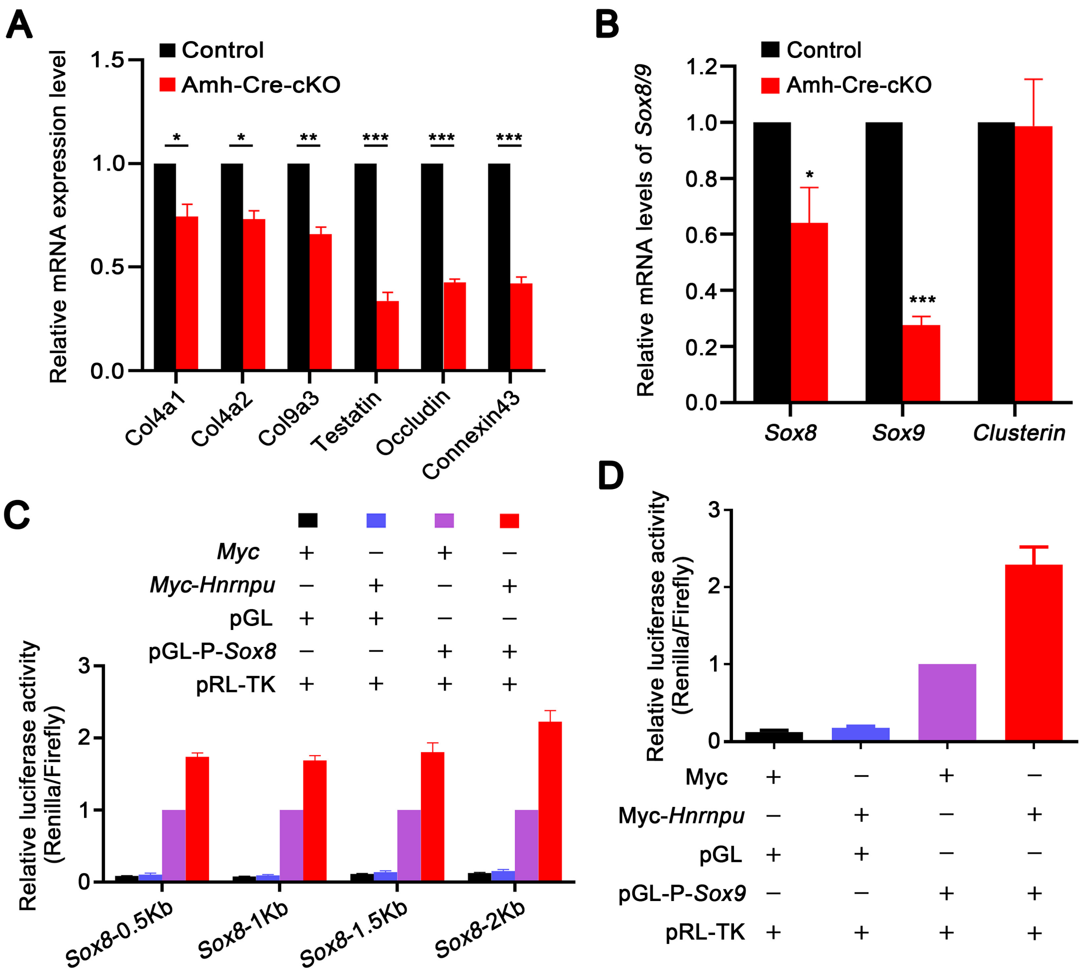

Figure S3. hnRNPU deficiency in Sertoli cells affects the gene transcription levels in Sertoli cells. (**A**) RT-qPCR analyses of expression levels of *Col4a1, Col4a2, Col9a3*, *Testatin, Occludin,* and *Connexin43* mRNAs in control and Amh-Cre-cKO testes at P3 are shown. Data are presented as mean ± SEM, n = 3. *$P < 0.05$, **$P < 0.01$, and ***$P < 0.001$ by Student's *t*-test. (**B**) RT-qPCR analyses show the expression levels of *Sox8*, *Sox9,* and *Clusterin* mRNAs in purified Sertoli cells from control and Amh-Cre-cKO mice at P3. Data are presented as mean ± SEM, n = 3. *$P < 0.05$, ***$P < 0.001$ by Student's *t*-test. (**C-D**) Luciferase-based reporter assays showing the luciferase activity of the different *Sox8* promoter regions (C) and *Sox9* promoters (D) in HEK293T cells significantly increase when hnRNPU is overexpressed.

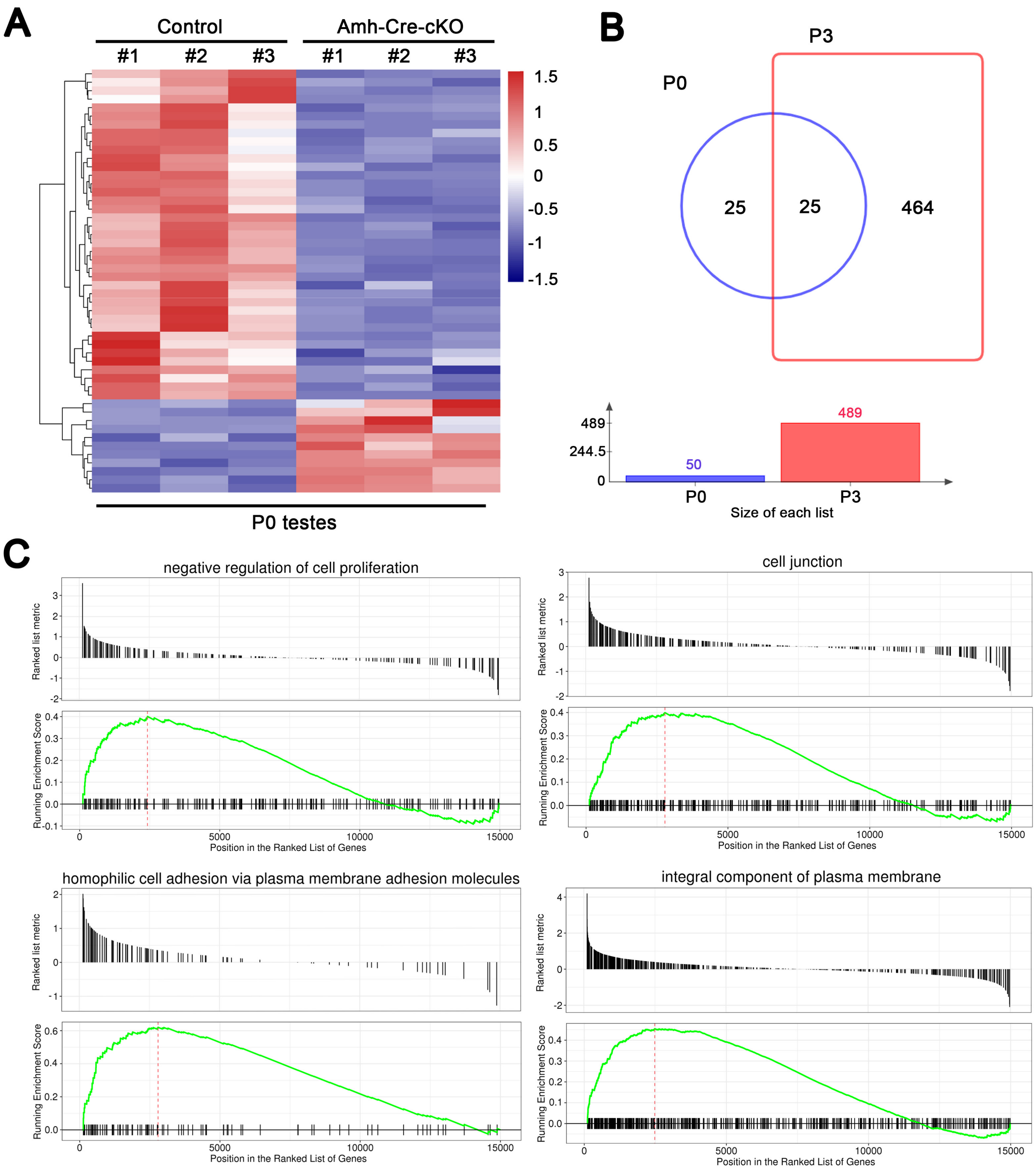

Figure S4. Loss of hnRNPU in Sertoli cells slightly alters genome-wide transcriptome profiles at P0 testes. (**A**) Heat-map shows the differential expression genes (DEG) in control and Amh-Cre-cKO testis at P0. (**B**) Venn diagram showing the numbers of dysregulated genes between P0 and P3 Amh-Cre-cKO testis. (**C**) Graph showing gene set enrichment analysis (GSEA) of RNA-seq data at P3 testes. Amh-Cre-cKO testes show an overrepresentation of genes related to cell proliferation, cell junction, and adhesion functions.
